# Surface-Modified Silica Hydrogels for the Programmable Release of Bisphosphonate Anti-Osteoporosis Drugs: The Case of Etidronate

**DOI:** 10.3390/ma16093379

**Published:** 2023-04-26

**Authors:** Fanouria-Eirini G. Alatzoglou, Maria Vassaki, Kalliopi Nirgianaki, Eleftherios Tripodianos, Petri Turhanen, Konstantinos D. Demadis, Konstantinos E. Papathanasiou

**Affiliations:** 1 Crystal Engineering, Growth and Design Laboratory, Department of Chemistry, University of Crete, 71003 Heraklion, Crete, Greece; fani06397@gmail.com (F.-E.G.A.); vassakimar@gmail.com (M.V.); kallia.niryanakis@gmail.com (K.N.);; 2 Biocenter Kuopio, School of Pharmacy, University of Eastern Finland, P.O. Box 1627, 70211 Kuopio, Finland; petri.turhanen@uef.fi; 3 Department of Chemistry, School of Sciences and Engineering, University of Wolverhampton, Wulfruna Street, Wolverhampton WV1 1LY, UK

**Keywords:** silica gels, hydrogels, drug release, bisphosphonates, osteoporosis, controlled release

## Abstract

Bisphosphonate drugs constitute the primary treatment for bone diseases such as Paget’s disease and osteoporosis. Despite their effectiveness, they also exhibit severe drawbacks, such as rapid excretion and limited oral bioavailability. High doses are usually administered to counterbalance these drawbacks. Subsequently, side effects are triggered, such as osteonecrosis of the lower jaw and esophageal cancer. Controlled drug release systems may be viable candidates to overcome those issues. Herein, we present novel functionalized silica-based hydrogels loaded with the osteoporosis drug etidronate (1,1-hydroxyethylidene-diphosphonate) used to control the release profile of the drug. Various methodologies were evaluated to control the initial release rate and the final released concentration of the drug. These included the gel density, by systematically increasing the initial concentration of silicate used to prepare the hydrogels, the presence of metal cations (Ca^2+^ and Cu^2+^), and the internal surface functionalization of the gel with silane-based grafting agents (with anionic, cationic, and neutral groups). This study also contributes to our continuous effort to develop new a priori programmable drug-loaded gels for the controlled release of osteoporosis drugs.

## 1. Introduction

Malignant bone tumors and osteoporosis are two of the most common skeletal dis-orders in humans [1,2]. The musculoskeletal system provides support to the human body and, as a result, bone disorders may significantly affect a patient’s daily life. Studies and statistics on bone diseases reveal that more than 200 million patients suffer from osteoporosis, while the disease causes almost 9 million bone fractures annually [3,4]. On top of that, patients suffering from other diseases, such as prostate cancer, breast cancer, lung cancer, and multiple myeloma, are also at high risk of developing bone diseases [5].

**Figure 1:**
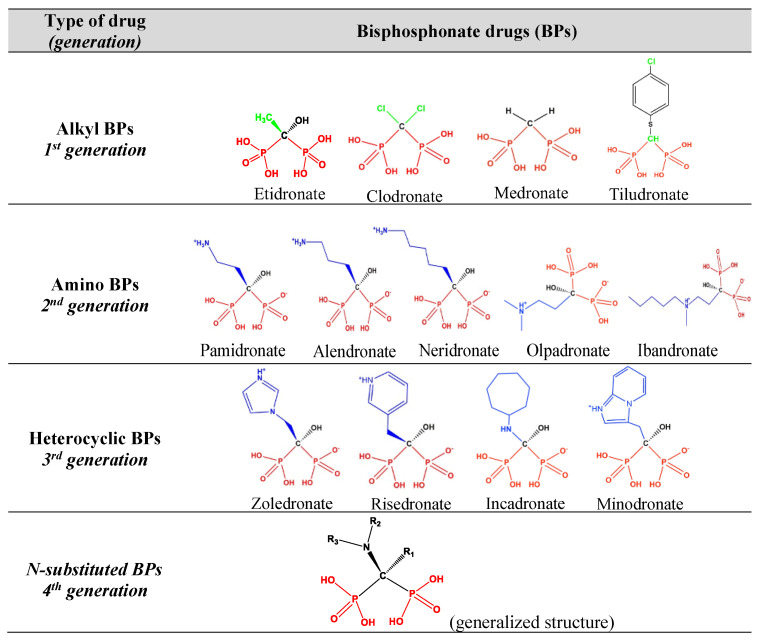
Examples of frequently administered BPs (according to generation) for the treatment of bone diseases.

Since 1977, bisphosphonates (BPs) have been used to treat bone diseases such as bone cancer, Paget’s disease, and osteoporosis, the last being the most prominent [6]. Some examples of BPs are shown in Figure 1.

Several are commercially available and are easily identifiable by the “-dronate” suffix (e.g., etidronate, pamidronate, zoledronate) in their commercial name. BPs can be envisioned as structural analogs of inorganic pyrophosphate. They are taken up by mature osteoclasts and inhibit farnesyl pyrophosphatase synthase, an enzyme of the mevalonate pathway [7]. They successfully inhibit bone resorption by inducing osteoclast apoptosis and thus, prevent the deterioration of bone microarchitecture [8]. An excellent example of the widespread use of BPs is the current treatment protocols for postmenopausal women at high risk of bone fractures. It is proven that alendronate (ALE), ibandronate (IBA) and risedronate (RIS) significantly reduce the risk of such fractures [9,10]. Despite the wide use of BPs, patients under such treatment suffer from several side effects. Some of them are osteonecrosis of the jawbone, esophageal cancer, ocular inflammation, and atrial fibrillation [11,12,13].

This notwithstanding, other disadvantages include their fast excretion and limited oral bioavailability (<6%). Subsequently, high dosages of medication are needed for BPs to achieve their therapeutic effect [14]. Recent studies have also raised concerns that BPs can adversely affect glucose metabolism [15,16]. Therefore, ongoing research on BPs is targeted to discover new treatment protocols based on controlled release processes to overcome some of the above disadvantages. Functionalized substrates, such as hydrogels that can not only host the loaded drug, but can also allow its controllable release, could be potential candidates in such processes [17,18]. However, in some cases, the drug exhibits low solubility in biological fluids [19] or is metabolized rapidly [20].

Only a few examples of BP controlled release systems have been published in the literature. The first was discovered in 2006 by Wang et al. and was based on a polymeric chain containing grafted PAM [21]. Huang et al. presented another release system based on microspheres of polymer-mediated, ALE-loaded hydroxyapatite [22]. A system that combined nanoparticles of poly(lactide-glycolide) acid, poly(ethylene glycol), and zoledronic acid (ZOL) was evaluated by Chaudhari et al. [23]. Moreover, Kim et al. prepared calcium phosphate microspheres that controlled the release rate of ALE [24]. Finally, Johnson et al. studied the release of ETID in vitro using refillable polyurethane reservoirs [25]. In recent years, BP controlled release systems have appeared in which the “active” BP is part of a coordination polymer backbone. When exposed to appropriate conditions, these metal-bisphosphonate systems affect the controlled release of the BP. Almeida Paz et al. have used metal ALE coordination polymer-based systems [26], and Demadis et al. have studied several 1st and 2nd generation metal-BP systems [27,28]. Colloidal/amorphous silica has been thoroughly studied as a host for active agents and has been proven as a non-toxic material [29,30,31,32,33]. Whether in the form of xerogels, mesoporous silica, or fumed silica nanoparticles, silica is employed in the food industry and the pharmaceutical sector. More specifically, mesoporous silica nanoparticle systems have shown promising results as multi-purpose drug hosts [34]. Nevertheless, the fabrication processes of such systems require special conditions, such as high temperature and pressure or extreme alkaline or acidic environments. Unfortunately, such conditions add extra costs and additional experimental steps in the fabrication process [35]. Interestingly, Balas et al. studied two hexagonally ordered mesoporous materials, MCM-41 and SBA-15, as substrates for BP adsorption and release [36].

This paper builds upon our earlier studies on easy-to-prepare “pure” silica hydrogel-type controlled release systems [37,38,39]. We previously showed that pristine silica-based hydrogels can host and incorporate a wide variety of BPs and subsequently release them in a controlled manner. Hence, herein we investigate new methodologies to control the release (the delay in the drug release is typically desirable) of the hydrogel-embedded drug ETID by exploring three distinct strategies: (a) the modification of hydrogel density, (b) the use of BP-binding metal cations, and (c) the modification of the gel’s internal surfaces with grafting functionalized (cationic, anionic, or neutral) silane agents. To our knowledge, such chemical modifications of silica hydrogel systems, which profoundly influence drug release, have not been reported in the literature. The reported materials are designed for the oral administration route; hence, the release studies were performed at a low pH (~3) that mimics that of the human stomach.

## 2. Materials and Methods

### 2.1. Materials

Sodium metasilicate pentahydrate (Na_2_SiO_3_·5H_2_O, “silicate”, from here on), NaOH 2M, CaCl_2_·2H_2_O, and CuCl_2_·2H_2_O were purchased from Merck KGaA (Darmstadt, Germany). ETID (in its acid form) was received from Solutia Inc. (St. Louis, MO, USA) as a 60% solution (Dequest 2010). APTES ((3-aminopropyl)triethoxysilane, 98%) and CPTS ((3-chloropropyl)trimethoxysilane, 97%) were purchased from Alfa Aesar (Kandel, Germany). TESPSA (3-(triethoxysilyl)propylsuccinic anhydride, 97%) was purchased from Fluorochem Ltd. (Hadfield, UK). Deuterium oxide (99.9% D with 0.05% 3-(trimethylsilyl)propionic-2,2,3,3-d4 acid, sodium salt, TSP) and HCl 37% were also purchased from Merck KGaA (Darmstadt, Germany). Deionized (DI) water from an in-house laboratory ion-exchange resin was used in all experiments. For consistency, all gels were synthesized in high form borosilicate beakers (volume 250 mL, diameter 60 mm, height 120 mm), and the ETID release experiments from the synthesized gels were performed in the same containers.

### 2.2. Instrumentation

Analytical pipettes (Witeg Labortechnik GmbH, Wertheim, Germany) were used for solution sampling. A ΤwpH315i pH meter combined with a SeTix41 electrode was used whenever pH adjustment was necessary. Solid state NMR experiments were performed on a Bruker Avance 300 NMR spectrometer with a 7 mm MAS wide bore probe. The operating resonance frequency (rf) of 300.1 MHz was used for for ^1^H, 59.6 MHz for ^29^Si, and of 121.5 MHz for ^31^P measurements. Zirconia rotors of 7 mm size, with hotmKEL-F inserts, were used. For direct excitation measurements π/2 pulses at rf fields of 34.7 kHz on ^29^Si and 65.8 kHz on ^31^P were applied. The interscan delay was set to 180 s for ^31^P and 120 s for ^29^Si. During acquisition, TPPM decoupling with a rf field at 50.0 kHz was used. The measurements were performed at a sample spinning speed of 7 kHz. For the ^1^H-^31^P CP MAS experiments, a ramped CP using a rf field of 53.2 kHz for the spinlock on ^31^P, an 80–100% ramp on the proton channel, and a contact time of 1.5 ms were applied. The corresponding decoupling was performed using the TPPM decoupling scheme at a rf field of 50.0 kHz during acquisition. The interscan delay was set to 5 s. An AVANCE 300 (Bruker, Karlsruhe, Germany) spectrometer was used for the release experiments. EDX data was collected with a JOEL JSM-6390LV electron microscope.

### 2.3. Preparation of ETID-Loaded Silica Hydrogels

Experimental conditions, such as temperature (ambient), acids/bases used for pH adjustment, type of glass beaker used, and ETID quantity (0.850 mmol, 165 mg as ETID acid), were the same for every gel.

The basic procedure is described below for the control gel. This is a gel that is used as a standard, and all drug release comparisons are made against this gel. It can also be appropriately modified to incorporate other components (see following sections). DI water (10 mL) was added into a borosilicate (high form) glass beaker (250 mL). A quantity of silicate (0.66 g, 3.14 mmol) was added to it, along with 0.25 mL of ETID (of the 60% stock solution, 0.850 mmol), under rigorous stirring. At this point, the solution pH was ~12.5. The pH was lowered to ~7.0 by using 0.32 mL of a concentrated HCl solution (37%). The magnetic stirring bar was removed immediately, leaving the system to rest. The onset of gel formation was within 10 min, but the freshly formed gel was allowed to mature for 12 h, after which a shapely and translucent gel formed. The preparation of gels with various densities was possible with the addition of appropriate amounts of silicate (see Table S1). The lowest and highest silicate that resulted in the tractable gels were 0.333 g (3.33% *w*/*v*) and 1.600 g (16% *w*/*v*), respectively.

### 2.4. Preparation of Hydrogels Incorporating Ca^2+^ and Cu^2+^ Ions

The Ca^2+^-loaded gels were prepared as follows. DI water (10 mL) was added into a beaker (250 mL). A quantity of silicate (0.66 g, 3.14 mmol) was then added, along with 0.25 mL (of the 60% solution, 0.850 mmol) of ETID, under rigorous stirring. At this point, the solution pH was ~12.5. The pH was then lowered to ~1.3 using 0.600 mL of concentrated HCl (37%), and a quantity of CaCl_2_·2H_2_O was added, depending on the desired Ca:ETID molar ratio. Gels with two different Ca:ETID molar ratios, 1:4 and 1:2, were prepared, which required the addition of 37 and 75 mg of CaCl_2_·2H_2_O, respectively. The pH was readjusted to ~7 with NaOH 2M after the addition of the Ca^2+^ source. Further details can be found in Table S2.

The Cu^2+^-loaded gels were prepared as follows. DI water (10 mL) was added into a beaker (250 mL). A quantity of silicate (0.66 g, 3.14 mmol) was then added, along with 0.25 mL (of the 60% solution, 0.850 mmol) of ETID under rigorous stirring. The pH was adjusted to ~1.3 using 0.600 mL of concentrated HCl (37%) and a quantity of CuCl_2_·2H_2_O was added, depending on the desired Cu:ETID molar ratio. Gels with five different Cu:ETID molar ratios were prepared, 1:1, 1:2, 1:3.2, 1:6.5, and 1:13, which required the addition of 174, 87, 53.5, 26.7, and 13.4 mg of CuCl_2_·2H_2_O, respectively. The pH was readjusted to ~7 with NaOH 2M after the addition of the Cu^2+^ source. Further details can be found in Table S3.

### 2.5. Preparation of Silane-Grafted Hydrogels

**Figure 2:**
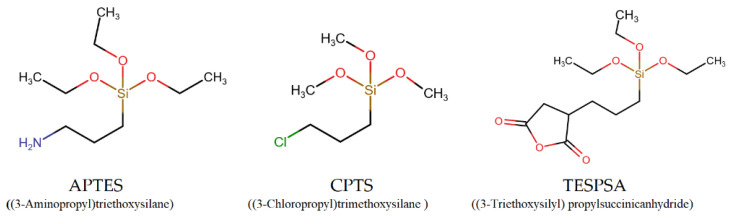
Schematic structures of the three silane-based grafting agents.

The silane-grafted hydrogels were prepared in a similar manner, with the difference that the dissolution of silicate was followed by the addition of the silane grafting agent, and that the gel maturation time was 80 h. The reasons for the extended maturation period will be discussed later. Further details on the preparation of the silane-grafted hydrogels can be found in Tables S4–S6. The schematic structures of the three silane agents used are shown in Figure 2.

The APTES-grafted gels were prepared as follows. DI water (9.33 mL) was added into a glass beaker. A quantity of silicate (0.66 g, 3.14 mmol) was then added under stirring until its complete dissolution, followed by the addition of 74.8 μL APTES, an amount that corresponds to an APTES:silicate 1:10 molar ratio. Subsequently, ETID (0.25 mL of the 60% solution, 0.850 mmol) was added under stirring. The pH was adjusted to 6.9 using 0.340 mL of concentrated HCl (37%). The stirring bar was then removed immediately, and the solution was allowed to stand for 80 h for the gel to form and mature.

The CPTS-grafted gels were prepared as follows. DI water (9.37 mL) was added into a glass beaker. A quantity of silicate (0.66 g, 3.14 mmol) was then added under stirring until its complete dissolution, followed by the addition of 57.5 μL CPTS, an amount that corresponds to a CPTS:silicate 1:10 molar ratio. Subsequently, ETID (0.25 mL of the 60% solution, 0.850 mmol) was added under stirring. The pH was adjusted to 7.20 using 0.320 mL of concentrated HCl (37%). The stirring bar was removed immediately, and the solution was allowed to stand for 80 h for the gel to form and mature.

The TESPA-grafted gels were prepared as follows. DI water (9.38 mL) was added into a glass beaker. A quantity of silicate (0.66 g, 3.14 mmol) was then added under stirring until its complete dissolution, followed by the addition of 44 μL TESPSA, an amount that corresponds to a TESPA:silicate 1:20 molar ratio. Subsequently, ETID (0.25 mL of the 60% solution, 0.850 mmol) was added under stirring. The pH was adjusted to 6.90 using 0.320 mL of concentrated HCl (37%). Subsequently, ETID (0.25 mL of the 60% solution, 0.850 mmol) was added under stirring. Importantly, higher TESPA:silicate molar ratios did not result in gel formation.

### 2.6. General Drug Release Protocol

The above-mentioned gel preparation methods yielded shapely and translucent gels. After the maturation period required for each case, the gel was used to set up the ETID release protocol. First, pre-acidified (pH~3) DI water (50 mL) was carefully added on top of the solidified gel. This marked t = 0 of the release experiment. The gel/water system was left under quiescent conditions (not stirred). Aliquots (0.350 mL) of the supernatant aqueous phase were withdrawn (via a high-precision pipette) every hour for the first 6 h. Two aliquots were withdrawn at the 9th and at the 12th hour. Finally, three final aliquots (at the 24th, 30th and 48th hour) were collected. Each aliquot was mixed with 0.150 mL of D2O (99.9 atom% D) that contained 0.05 wt.% (4.3375 μmol) 3-(trimethylsilyl)propionic-2,2,3,3-d4 acid, sodium salt, TSP) as an internal quantification standard. Quantification of the ETID concentration in each sample was achieved by peak (-CH_3_) integration in the ^1^H NMR spectrum (at 293.2 K) and its comparison to the peak of the TSP standard solution peak [-Si(CH_3_)_3_]. Figure S1 presents an ^1^H NMR spectrum as an example of the integration performed over the ETID and TSP peaks. Respectively, the recorded spectrum of each aliquot was used to quantify the amount of ETID during the sampling process. The relative peak integrals obtained were recalculated to produce release curves that plot % drug released vs. time (in hours).

### 2.7. Kinetic and Statistical Analysis

#### 2.7.1. Sink Conditions

According to the definition of “sink condition”, for drug dissolution results to be meaningful, the volume of the dissolution medium must usually be about 5 to 10 times greater than the volume present in the saturated solution of the targeted chemical. In this case, the maximum solubility of ETID is 700 g/L (700 mg/mL). Since the volume of the supernatant aqueous phase used in our experiments was 50 mL, the ETID solubility is calculated to be 35 g (in 50 mL). The ETID mass in all the gels used was 165 mg, which could potentially dissolve in 50 mL. Hence, the amount of ETID used is 35/0.165 = 212 below the solubility limit of ETID, therefore, our experiments are consistent with “sink conditions”.

#### 2.7.2. Repeatability and Reproducibility of the Release Experiments

**Figure 3:**
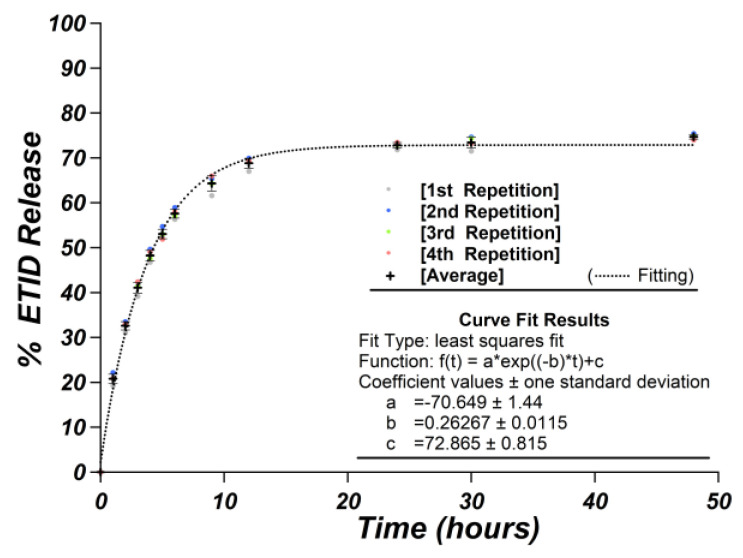
Release curves of four identical control gels, including error bars.

The release curves obtained from the quadruplet control experiments are depicted in Figure 3. The average value of each set of points was calculated and used, respectively, to extract the standard deviation (σ) of the % release. The sigma varies from 0.50 to 1.68, which signifies a quantification process of high accuracy (values available in Table S7). The use of ^1^H NMR, as a technique for BPs quantification, was intensely evaluated by our group, and previous studies showed that the error margin is very narrow [27,28,37]. The precision of this method is consistent with other quantification studies in food samples using ^31^P NMR spectroscopy [40].

Due to the large number of release experiments, samplings, and spectra acquisitions, and the established high accuracy of the data obtained, we decided that running quadruplets for each release experiment was not practical. Hence, we assumed the worst σ value (1.68) and applied it to all data. To avoid outliers, occasional experiments were duplicated, and the obtained results always fell within the assumed standard deviation.

#### 2.7.3. Calculation of Initial Rates

The initial rate is defined as the amount of drug released per unit time at the initial stages of the release experiment, where the curve shows linearity. We selected the first three hours of the release experiment and noted that there is an acceptable linear behavior (R^2^ > 97%). The initial rate herein is expressed in μmol/min, and it represents a good tool for comparing the various gel systems studied.

#### 2.7.4. Kinetic Models

Several mathematical models have been proposed for the statistical analysis of drug release data, which are usually based on non-linear equations. Statistical programs such as GraphPad Prism, Sigmaplot, etc., can be used for the non-linear fitting of dissolution data, but require manual definition of the equations and initial values of each parameter. For the kinetic study of the ETID release from the control hydrogels, the DDSolver application was used, which is a plug-in for Microsoft Excel and contains 40 different built-in dissolution models for modeling the drug release data [41]. The evaluation of the kinetic models was carried out by comparing three statistical parameters: (a) the coefficient of determination (R^2^), (b) the model selection criterion (MSC), and (c) the Akaike information criterion (AIC). Specifically, the most suitable kinetic model for the dissolution data contains the highest R^2^ and MSC values, as well as the lowest AIC values.

## 3. Results

### 3.1. Gel Density: Impact of Silicate Concentration on the ETID Release Profile

**Figure 4:**
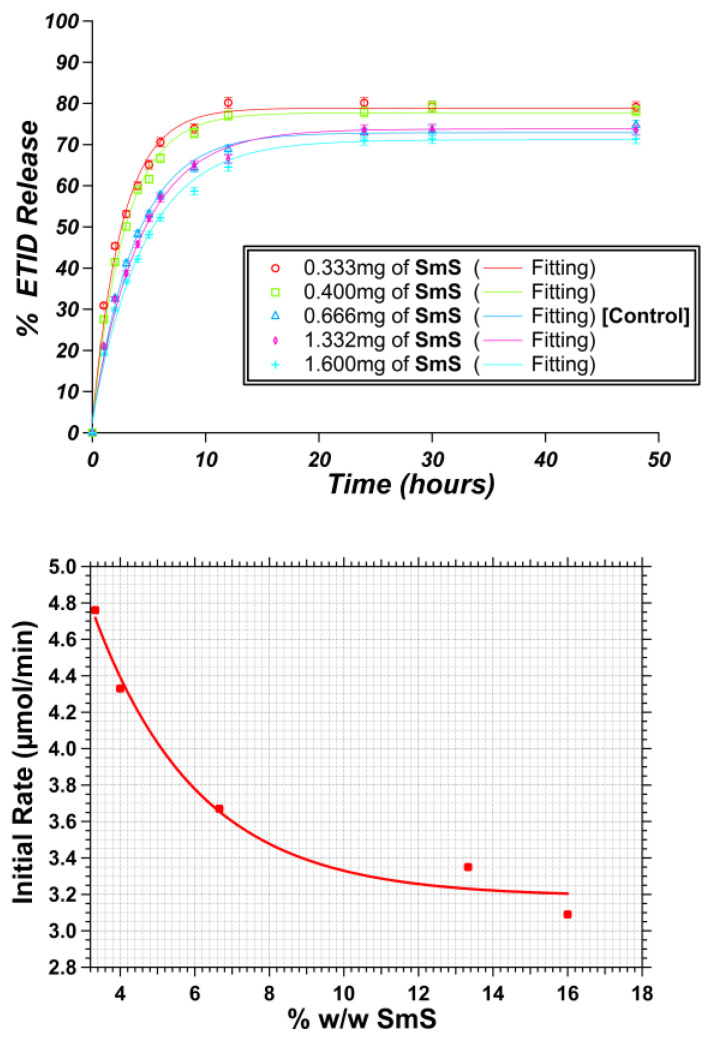
(**Upper**): Controlled release curves of five silica hydrogels with systematically different silicate content. (**Lower**): The relationship between the average initial rate and the silicate content (SmS = sodium metasilicate).

A study was conducted to explore how systematically different concentrations of silicate used in gel synthesis influenced the release profile of ETID. Five different gels were synthesized using silicate concentrations ranging from 3.3% up to 16% *w*/*w* (Table S1). Importantly, we could not synthesize stable and robust hydrogels with a silicate content lower than 3.3% *w*/*w*, or higher than 16% *w*/*w*. The obtained ETID release profiles are presented in Figure 4. The studies revealed that as the silicate concentration is lowered, a more rapid release and a higher plateau value (in %) of ETID are observed. The data obtained during the first 3 h corresponded to a relatively linear part of the curves (Figure S2) and were used to obtain the initial rate values. Figure 4 shows the relationship between the silicate concentration and the initial rate.

**Figure 5:**
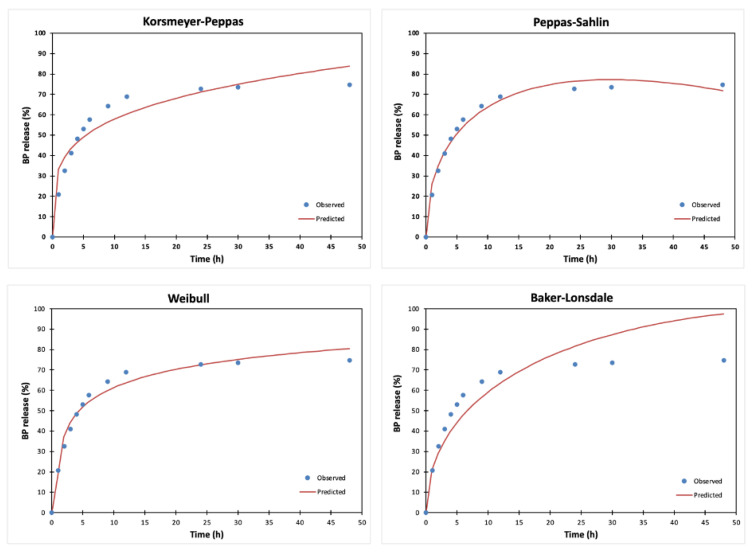
Observed data of ETID release from a control hydrogel, depicted with the kinetic model prediction using Korsmeyer–Peppas, Peppas–Sahlin, Weibull, and Baker–Lonsdale models. These kinetic models present better statistical parameters (r^2^, AIC, MSC) than the others.

We evaluated several known kinetic models for fitting the results of the drug release from hydrogels, including zero-order, first-order, Higuchi’s, Korsmeyer–Peppas, Hixson-Crowell, Peppas–Sahlin, Hopfenberg, Baker–Lonsdale, and Weibull models to explain the pathway of drug release, and the results are presented in Table 1 and Figure 5. The average value of ETID release from the control hydrogels is better described by the Peppas–Sahlin model because this kinetic model presents the highest determination coefficient r^2^ and MSC values, in combination with the lowest AIC value. This kinetic model expresses the release of drugs from polymeric devices, and it is a combination of two mechanisms, Fickian diffusion and relaxation (Case II). The term (k_PS_∙t^m^) of the equation indicates the Fickian diffusional contribution, and the term (k^2^∙t^(2∙m)^) represents the Case II—relaxational contribution, respectively [42]. Based on the similarities of the release curves from all systems described in our paper, we have treated all data according to the Peppas–Sahlin model.

### 3.2. Metal-Loaded Hydrogels

These gels were designed to take advantage of the general property of bisphosphonates to strongly bind to metal cations [43]. Their binding ability generally follows the order M^4+^ > M^3+^ > M^2+^ > M^+^. Two divalent metals were selected, Ca^2+^ and Cu^2+^. The affinity of ETID towards some common divalent cations follows the trend (log*K* values in parentheses) Cu^2+^ (11.8) >> Mg^2+^ (7.3) > Ca^2+^ (6.4) > Zn^2+^ (5.1) [44]. Ca^2+^ was chosen because it is a non-toxic, widely abundant metal ion that is used in drug formulations and dietary supplements. Cu^2+^ is beneficial in trace amounts but is generally considered a toxic element for living organisms. Its use in our gels does not imply any intent for its use in pharmaceutical formulations, but merely as a laboratory “tool” for ETID binding and immobilization. The idea of evaluating the impact of Ca^2+^ and Cu^2+^ on the release profile of ETID was based on the premise that the metal ions will coordinate to the ETID molecules, immobilize them inside the gel and thus, decelerate the drug release by decreasing both the initial rates and plateau values.

#### 3.2.1. Ca^2+^-Loaded Hydrogels

**Figure 6:**
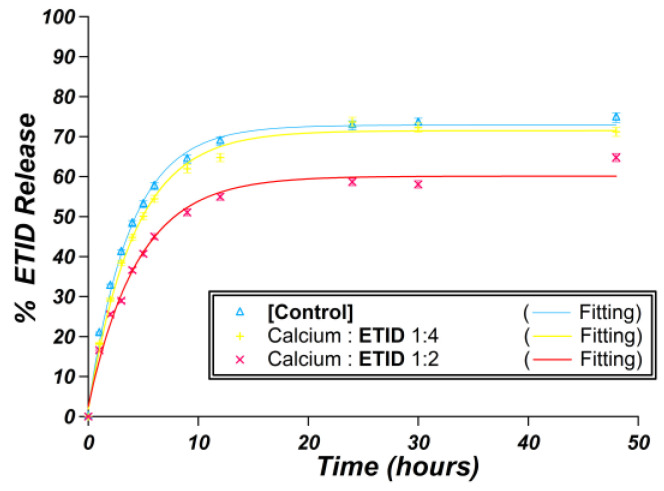
Control release curves for the control gel (blue), Ca:ETID 1:4 (yellow), and Ca:ETID 1:2 (red).

Calcium chloride was the source of the Ca^2+^, and the latter was incorporated during (and not after) gel formation. Two different Ca:ETID molar ratios were evaluated, 1:2 and 1:4, by changing the Ca^2+^ content, while the ETID content was the same as in the control (0.850 mmol). The pH in all cases was set to the value of 6.7. Interestingly, the release curves presented in Figure 6 reveal a small, but measurable, impact of Ca^2+^ on both the initial rate and the plateau value (Table S8). In both cases, the formation of a solid Ca-ETID precipitate was detected in the gel matrices. This precipitate was collected and characterized by FTIR and EDX spectroscopies (Figures S3 and S4). The available data are consistent with the formation of a previously reported Ca-ETID coordination polymer [45].

#### 3.2.2. Cu^2+^-Loaded Hydrogels

**Figure 7:**
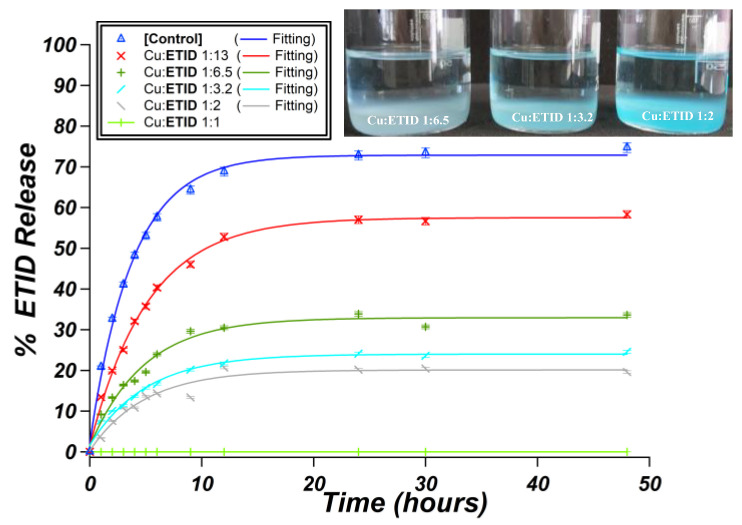
Release curves for the gels: control (blue), and the Cu^2+^-loaded gels with varying Cu:ETID ratios, 1:1 (light green), 1:2 (grey), 1:3.2 (turquoise), 1:6.5 (green), and 1:13 (red).

**Figure 8:**
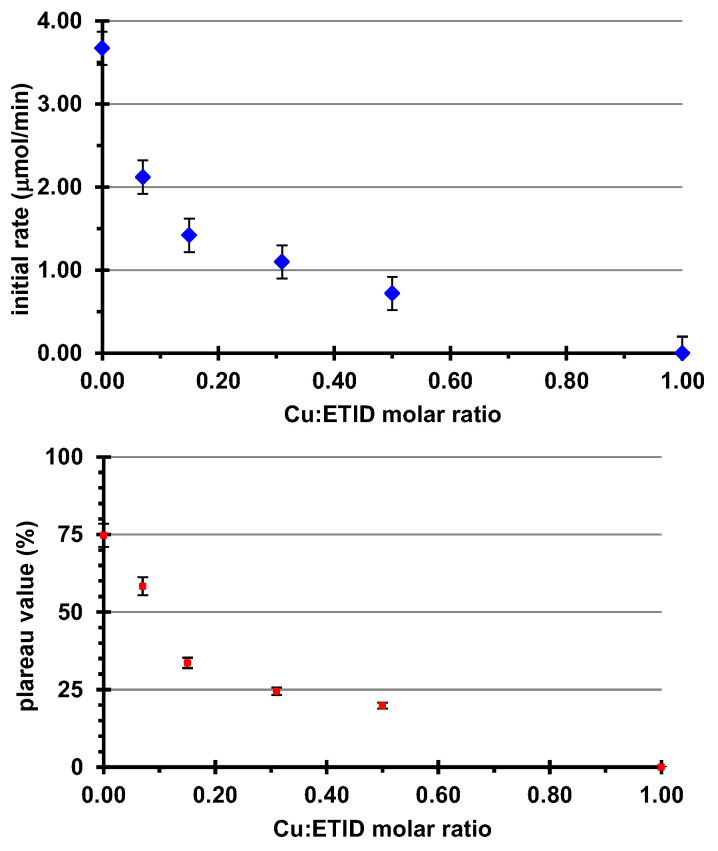
The effect of the Cu:ETID molar ratio on the initial rate (above) and the plateau values.

**Figure 9:**
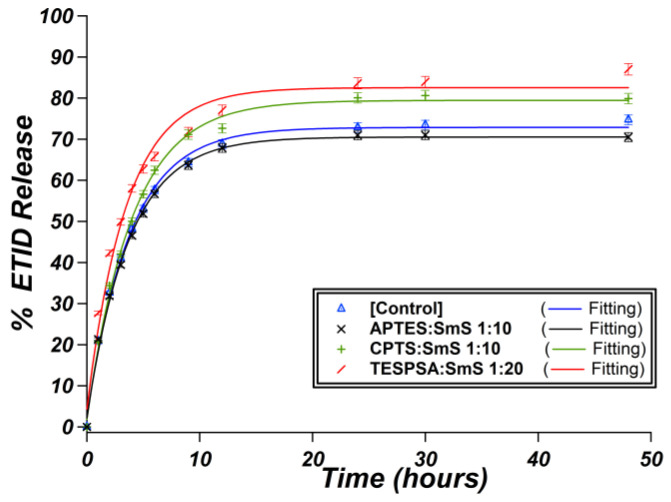
The release of ETID from hydrogels synthesized from three different grafted-drug loaded hydrogels: APTES:silicate 1:10 (black), CTPS:silicate 1:10 (green), TESPSA:silicate 1:20 (red), and control (blue). SmS = sodium meta-silicate.

ETID is expected to show a much higher affinity towards Cu^2+^ (compared to Ca^2+^) [44]. Hence, in the case of the Cu^2+^-loaded gels, a stronger impact on initial rates and plateau values should be expected. Figure 7 presents the release curves of all Cu^2+^-loaded hydrogel systems studied. Initially, a gel with a Cu:ETID molar ratio of 1:1 was synthesized at the same pH value (pH~6.9). In this setup, the above ratio ensures charge balance (+2 from the Cu, and −2 from ETID), so complete binding of ETID by Cu^2+^ is expected. Interestingly, no ETID was detected in the supernatant aqueous phase during a 48-h experiment. This was a strong indication that all the ETID was coordinated by Cu^2+^ in the gel, quantitatively and irreversibly. Hence, systematically lower Cu^2+^ concentrations were tested, as shown in Figure 8 and Table S9. As the concentration of Cu^2+^ increases (the Cu:ETID molar ratio decreases), the initial rate and the plateau value systematically decrease. These profound changes are shown in Figure 9. Further elaboration on these results can be found in the Discussion Section.

These observations are consistent with the higher affinity of ETID for Cu^2+^ and reflect the tendency of Cu^2+^ to form a variety of coordination complexes and compounds with phosphonate ligands [46,47]. The above results proved that the presence of metal cations can be an inspiration for further programming the silica hydrogels in terms of obtaining different controlled release profiles.

### 3.3. Surface Functionalization of ETID-Loaded Hydrogels by Silane Agents

In this and previous studies [37] several experimental variables were altered (e.g., concentration of silicate, pH, identity of the drug (primarily the R side-group), temperature, presence of metal ions, etc.); however, these factors involved “pristine” silica hydrogels, in which the internal gel surface remained unaltered. This section explores how grafting on the gel’s internal pore walls can influence the release profile of ETID. Thus, three diverse silane grafting agents were evaluated. All release results obtained are shown in Figure 9.

#### 3.3.1. The Case of (3-Aminopropyl)triethoxysilane (APTES)

APTES (Figure 2) is one of the most common grafting agents frequently used for surface modification with alkoxysilane moieties [48] and for the adhesive bonding of semiconductor wafers [49]. The aqueous hydrolysis of the ethoxy groups in APTES results in the generation of a silanetriol moiety [-Si(OH)_3_], with the concomitant production of three equivalents of ethanol as by-product. The process can be easily monitored using both ^1^H and ^13^C NMR. The NMR data were collected 10 min after adding a certain amount of APTES in ~10 mL of water (solvent extracted). The absence of ethoxy-group signals in both ^1^H and ^13^C NMR studies confirms that the APTES is fully converted to the hydrolyzed silanetriol analog (Figures S5 and S6).

The next step would be to implement the hydrolysis of APTES and its subsequent grafting within the silica hydrogel. Initially, hydrogels were prepared in the presence of APTES with an APTES:silicate molar ratio of 1:10, as described in Section 2.5. We then measured the ethanol concentration following the same protocol used in the release studies. The ethanol release curve suggests that nearly 85% of APTES was hydrolyzed and subsequently grafted onto the surface of the silica gels (Figures S7 and S8). Finally, when APTES is added to the supernatant aqueous phase on top of an “empty” hydrogel (with no APTES), it is absorbed by the gel (~90% within 80 h, Figures S9 and S10). The presence of the grafted APTES within the gel was also confirmed by ^1^H Solid-State NMR data collected at TU-Dresden (Figure S11, signal of the propyl group at 0.72 ppm).

Drug release from an ETID-loaded APTES:silicate 1:10 gel showed only slight changes compared to the non-grafted drug-loaded hydrogel (control), Figure 9. The expectation was to observe a slower drug release, and perhaps a lower plateau value, based on the premise that the protonated –CH_2_CH_2_CH_2_NH_3_^+^ group of the APTES would induce an electrostatic “immobilization” of the negatively-charged ETID and thus, delay its release. The observed insensitivity of the grafted gel is undoubtedly related to the low APTES content within the gel. Unfortunately, stable and robust gels containing a higher concentration of APTES could not be fabricated.

#### 3.3.2. The Case of (3-Chloropropyl)trimethoxysilane (CPTS)

Another grafting agent tested was CPTS (Figure 2), containing three methoxy groups (which also undergo hydrolysis, just as do the ethoxy groups, within 10–20 min) and a chloro- substituent on the terminal C of the propyl side-chain. Similarly to APTES, the grafting process can be followed by the monitoring of methanol production. The presence of the electronegative chloro- substituent renders a partial negative charge (δ−) to the molecule. Hence, in contrast to the positively charged amine in APTES, it might be possible to observe the opposite effect on ETID release, i.e., a (small) acceleration.

Drug release from an ETID-loaded CPTS:silicate 1:10 gel showed only slight changes in the initial rate, but an increase of ~10 percentage units in the plateau value, compared to the control (no CPTS), Figure 9.

#### 3.3.3. The Case of (3-Triethoxysilyl)propylsuccinic Anhydride (TESPSA)

The third grafting agent tested was TESPSA (Figure 2). In this case, unfortunately, the fabrication of gels with a molar ratio of TESPSA:silicate 1:10 was not possible. After experimentation with various molar ratios, the TESPSA:silicate 1:20 molar ratio yielded a stable and robust gel (the pH was adjusted to ~6.9). Higher amounts of TESPSA produced unstable and “runny” gels. As before, the presence of the grafted TESPSA in the gel was confirmed by solid-state ^1^H NMR on a dried gel sample after water evaporation (Figure S12).

The backbone of TESPSA contains a succinic anhydride. Such anhydrides tend to undergo ring-opening in aqueous systems, especially in basic solutions, producing the “open” dicarboxylate moiety [50]. The highly alkaline silicate solution (before pH adjustment and the gel formation) offers the ideal environment for the ring-opening of the succinic anhydride. As a result, two carboxylate groups are generated during gel formation. The pH re-adjustment, which was needed for further gel formation and release studies, did not affect those negatively charged moieties. Both the FTIR and ^13^C NMR spectra clearly show the presence of the “opened” anhydride in an aqueous solution with pH~6.9. After solvent (water) evaporation, an FTIR spectrum was collected (Figure S13). A shift of the peak at 1861 cm^−1^ (asymmetric vibration of anhydride) to 1716 cm^−1^ (asymmetric vibration of carboxylate) was observed. Peak shifts in two regions (33–48 ppm and 173–180 ppm) were observed in the ^13^C spectra of water hydrolyzed TESPSA at pH~6.9 and confirmed the formation of the carboxylate groups (Figure S14).

Drug release from an ETID-loaded, TESPSA-grafted gel (TESPSA:silicate 1:20) showed a small increase in the initial rate and an increase of ~15 percentage units in the plateau value, compared to the control (no TESPSA), Figure 9. We assign these changes to the presence of the anionic dicarboxylate moiety of TESPSA in the gel that develops repulsive ionic interactions with the negatively charged phosphonate moieties of ETID.

## 4. Discussion

**Figure 10:**
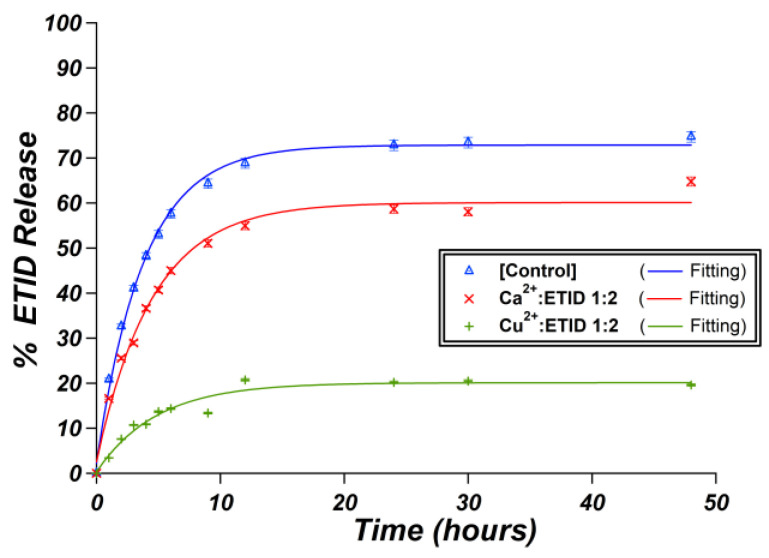
Comparative release curves of ETID from different gels: control (blue), Ca-loaded (red), and Cu-loaded (green).

The addition of divalent metal cations (Ca^2+^ and Cu^2+^) in the hydrogels drastically affected the release profile of ETID, suggesting that its affinity for divalent cations governs its release from the silica hydrogels. It appears that the higher the affinity, the lower (and slower) the release of ETID becomes. This can be visualized in Figure 10, where we compare the release profiles of identical hydrogels, which differ only in the metal cation incorporated. Both systems, the Ca^2+^- and the Cu^2+^-loaded hydrogels, were synthesized in the presence of the same molar concentration of metal cations. Both the initial rate and the plateau value are drastically affected. The Cu^2+^-loaded gel exhibits a ~5 times slower release and a more than 3 times decrease in the plateau value, compared to the Ca^2+^-loaded gel. Table S10 presents the relevant data.

In the case of Ca^2+^-loaded gels, the EDX spectrum of the precipitate formed on the surface of the gel suggested a Ca:P ratio of 1:1. Since ETID is a bisphosphonate, each ETID binds two Ca^2+^ cations. The control gels (gels with the same amount of ETID and silicate, at similar pH and volume) always retain ~25% of the ETID in the gel, as confirmed by consecutive release experiments using “fresh” supernatant [37]. Theoretically, we expected that the presence of Ca^2+^ would decrease the plateau value by 1/4. We also expected a decrease of 7/8 in the case of the Ca:silicate 1:4 system. That, however, was not the case. This result is ascribed to the pH value of the release experiment (~6.7). It is slightly lower than the pH value in which a neutral Ca-ETID “complex” forms. Logically, an amount of the complex remained diluted (and detectable by the NMR) in the supernatant during the release experiment. In the case of Cu^2+^, the gels acquired the familiar blue color, but there was no solid Cu-ETID complex formed for further characterization (see inset of Figure 7).

An alternative explanation of the observed lower initial rates and plateau values in the metal-loaded systems could be the following. The added divalent metal cations create precipitates within the gel pores, so the observed release is due to the “free” (uncoordinated) ETID molecules. This scenario, however, is not valid because of the reasons provided below. (a) Metal-ETID precipitation is only observed in the Ca-loaded gels, but not in the Cu-loaded gels. (b) If the observed ETID release was due to the “free” (metal unbound) ETID, similar (if not identical) values for the initial rate should be observed. This was not the case, as all metal-loaded systems exhibit consistently lower initial rate values. (c) The observed plateau values in the metal-loaded systems should correspond to the final release of the “free” (metal unbound) ETID. Again, this is not the case. For example, in the case of the Ca:ETID 1:2 system (Figure 6), and taking into account that each ETID binds two Ca^2+^ (i.e., two ETID molecules should bind four Ca^2+^ ions), ¾ of the ETID molecules should be “free”, and the expected plateau value would be expected to be around 56%. Although this is close to the plateau value observed, it cannot explain the 30% reduction in the initial rate value as compared to the control. This argument is further supported by the Cu:ETID 1:2 system. With the assumption that each ETID binds two Cu^2+^ (i.e., two ETID molecules should bind four Cu^2+^ ions, just as in the Ca system), ¾ of the ETID molecules should be “free”, and the expected plateau value would be expected to be around 56%, but it is measured to be ~20%.

Based on the available data, it seems that silica-based hydrogels can be a priori programmed to release the desired amount of ETID. This is important from a practical point of view because the concentration of the metal ion can be easily adjusted, and a variety of stable and robust hydrogels can be accessed via reliable synthetic protocols.

**Figure 11:**
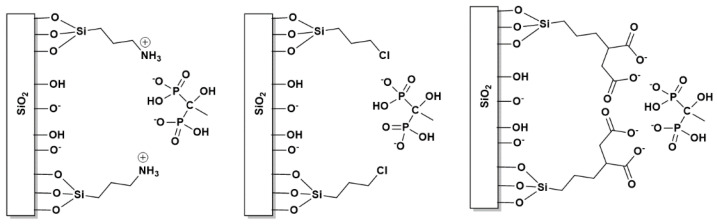
Schematic representations of interactions between ETID and the silica surfaces modified with different grafting agents: (**left**) APTES, (**middle**) CPTS, and (**right**) TESPSA.

APTES endows a local positive charge due to the –NH_3_^+^ group on its backbone. On the other hand, CTPS carries a Cl-substituent, and as a result, repulsion phenomena can occur. We also studied TESPSA as a grafting agent, which has a dicarboxylate structure under these conditions, with two negative charges (Figure 11).

APTES did not affect the release curve of ETID, even though slight differences were observed. One reason for this may be its low content in the gel, as mentioned above. In addition, it is reasonable to assume that the positive charge of the –NH_3_^+^ group, which would be expected to delay the release of ETID, is off-set (at least partially) by the neighboring deprotonated silanol groups. One can envision –Si–O^−^···^+^H_3_NCH_2_CH_2_CH_2_- “intramolecular” interactions taking place on the internal surfaces of the gel. Hence, any attractive electrostatic interaction between the negatively-charged ETID and the –NH_3_^+^ group from APTES are compromised, and therefore, the release rate is particularly unaffected.

On the other hand, we expected CTPS to generate weak repulsive electrostatic interactions with the anionic ETID molecules. Indeed, after the 3rd hour of the release, significant differences were noted. The ETID % final release of the CTPS-grafted hydrogel is lower than those of the APTES-grafted system. The values were also significantly different from the control gel. To further confirm that negatively charged grafts profoundly affect the release of ETID, we compared the results mentioned above with the curve of the TESPSA-grafted system. Even though the amount of TESPSA grafted to the gel is half that of the amounts of APTES and CPTS (however, the double negative charge on the carboxylate groups should be taken into account), significant differences were observed. These are attributed to the fact that there are two negative charges per TESPSA molecule. Moreover, TESPSA is much larger in size compared to the other grafting agents, so stereochemical effects might be expected, but it is presently unclear how these may affect the release rate.

## 5. Conclusions

In conclusion, this study reports the synthesis of modified silica-based hydrogels loaded with etidronic acid, a typical anti-osteoporotic therapy commercially known as Didronel^®^. Gel preparation does not require challenging conditions and can be carried out with cheap reagents. Three different strategies proved effective in the a priori functionalization of the gel matrices. The first revealed the impact of silicate concentration (gel density) on the ETID release profile. The higher the gel density is, the slower the release of ETID appears to be, with lower plateau values.

Moreover, incorporating divalent metal cations, such as Ca^2+^ and Cu^2+^, affected drug release. For example, by choosing the appropriate amount of Cu^2+^, gels with variable initial rates and plateau values were obtained. Interestingly, the addition into the gel of an equivalent molar amount of Cu^2+^ to that of ETID fully retained the latter in the gel matrix. The grafting processes in the gel’s interior with three silane grafting agents were also studied. The ETID release experiments also revealed that APTES, CPTS, and TESPSA played a significant role in further functionalizing drug-loaded silica hydrogels. Finally, the studies gels are injectable, an attribute that allows further administration routes in addition to oral delivery, such as local implants on bones [51]. Naturally, further studies regarding biocompatibility and mechanical stability are necessary. These characteristics are currently under study in our laboratory.

## Figures and Tables

**Table 1:** Release kinetics evaluation of the average ETID release from control hydrogels and calculated parameters for each equation.

Kinetics Models *	Evaluation Criteria			Parameters	
	r^2^	AIC	MSC		
**Zero-order** F = k_0_ ∙ t	−1.086	115.292	−1.454	k_0_ 2.430	-
**First-order**	0.632	94.467	0.282	k_1_ 0.130	-
F = 100 ∙ [1 − Exp(−k_1_ ∙ t)]					
**Higuchi** F = k_H_ ∙ t^0.5^	0.485	98.521	−0.056	k_H_ 15.290	-
**Korsmeyer–Peppas**	0.918	78.504	1.612	k_KP_ 33.514	n
F = k_KP_ ∙ t^n^					0.237
**Hixson-Crowell** F = 100 ∙ [1 − (1 − k_HC_ ∙ t)^3^]	0.380	100.734	−0.240	k_HC_ 0.029	-
**Peppas–Sahlin** F = k_PS(1)_ ∙ t^m^ + k_KPS(2)_ ∙ t ^(2 ∙ m)^	0.983	61.512	3.028	k_PS(1)_, k_PS(2)_ 28.899, −2.702	m 0.494
**Hopfenberg** F = 100 ∙ [1− (1 − k_HB_∙ t)^n^]	0.632	96.469	0.115	k_HB_ 0	n 5143.94
**Baker–Lonsdale** $\frac{3}{2}$[1 − (1 − $\frac{F}{100}$)^2/3^] − $\frac{F}{100}$= k_BL_ ∙ t	0.808	88.676	0.931	k_BL_ 0.008	-
**Weibull**	0.978	64.586	2.772	α, β 2.175, 0.329	Ti
F = 100 ∙ {1 − Exp[−((t − Ti)^β)/α^]}					0.890

* In all models, **F**: the percentage (%) of drug released at time t, **k_0_**: zero-order release constant, **k_1_**: first-order release constant, **k_H_**: Higuchi release constant, **k_KP_**: release constant incorporating structural and geometric characteristics of the drug-dosage form, n: diffusional exponent indicating the drug-release mechanism, **k_HC_**: Hixson–Crowell release constant, **k_PS(1)_**: Peppas–Sahlin release constant (related to the Fickian kinetics), **k_PS(2)_**: the constant related to Case-II relaxation kinetics, **m**: the diffusional exponent for a device of any geometric shape which inhibits controlled release, **k_HB_**: Hopfenberg release constant, n: 1, 2, and 3 for a slab, cylinder, and sphere, respectively, **k_BL_**: Baker Lonsdale release constant, **α**: the scale parameter which defines the time scale of the process, **β**: the shape parameter which characterizes the curve as either exponential (β = 1; case 1), sigmoid, S shaped, with upward curvature followed by a turning point (β > 1; case 2), or parabolic, with a higher initial slope and after that consistent with the exponential (β < 1; case 3), **Ti**: the location parameter which shows the lag time before the onset of the dissolution or release process and in most cases, will be near zero, **AIC**: Akaike information criterion, **r^2^**: determination coefficient, **MSC**: model selection criteria. Values shown in bold are better selections, according to the evaluation criteria.

## Data Availability

Data are available upon request.

## Supplementary Materials

The following supporting information can be downloaded at: https://www.mdpi.com/article/10.3390/ma16093379/s1, Further experimental details (tables with synthetic parameters), statistical data, NMR data (solution and solid), and gel characterization data.
