# Supplementary material for: Surface-Modified Silica Hydrogels for the Programmable Release of Bisphosphonate Anti-Osteoporosis Drugs: The Case of Etidronate
Source: Materials (Basel). 2023 Apr 26;16(9):3379. doi: 10.3390/ma16093379 (PMC10180253; doi:10.3390/ma16093379)
Supplement: Supplementary file 1 [file materials-16-03379-s001.zip › materials-2307435-supplementary.pdf]

materials

MDPI

Article

Surface-Modified Silica Hydrogels for the Programmable Release of Bisphosphonate Anti-Osteoporosis Drugs: The Case of Etidronate

Fanouria-Eirini G. Alatzoglou [1], Maria Vassaki [1], Kalliopi Nirgianaki [1], Eleftherios Tripodianos [1], Petri Turhanen [2], Konstantinos D. Demadis [1,*] and Konstantinos E. Papathanasiou [3,*]

[1] Crystal Engineering, Growth and Design Laboratory, Department of Chemistry, University of Crete, 71003 Heraklion, Crete, Greece; fani06397@gmail.com (F.-E.G.A.); vassakimar@gmail.com (M.V.); kallia.niryanakis@gmail.com (K.N.)

[2] Biocenter Kuopio, School of Pharmacy, University of Eastern Finland, P.O. Box 1627, 70211 Kuopio, Finland; petri.turhanen@uef.fi

[3] Department of Chemistry, School of Sciences and Engineering, University of Wolverhampton, Wulfruna Street, Wolverhampton WV1 1LY, UK

* Correspondence: demadis@uoc.gr (K.D.D.); k.papathanasiou@wlv.ac.uk (K.E.P.)

Table S1. The parameters of the synthesis for the set of gels with different concentrations of Sodium metaSilicate (**SmS**).

No	$Na_2SiO_3{\cdot}5H_2O$ (g)	DI H_2O (mL)	ETID (μL)	HCl 37% (μL)	pH
1	0.333	9.70	250	50	7.05
2	0.400	9.63	250	115	7.00
**3 (Control)**	**0.666**	**9.40**	**250**	**320**	**7.17**
4	0.800	9.29	250	446	7.00
5	1.332	8.90	250	850	7.11
6	1.600	8.67	250	1080	7.00

Table S2. The parameters of the synthesis for the set of gels with different [Ca^{2+}] concentrations and Ca:**ETID** ratios.

Ca^{2+}: ETID	$Na_2SiO_3{\cdot}5H_2O$ (g)	DI H_2O (mL)	ETID (μL)	HCl 37% (μL)	$CaCl_2.2H_2O$ (g)	pH
1:4	0.666	8.90	250	600	0.037	6.80
1:2	0.666	8.0	250	600	0.075	6.20

Table S3. The parameters of the synthesis for the set of gels with different [Cu^{2+}] concentrations and Cu:**ETID** ratios.

Cu^{2+}: ETID	$Na_2SiO_3{\cdot}5H_2O$ (g)	DI H_2O (mL)	ETID (μL)	HCl 37% (μL)	$CuCl_2{\cdot}2H_2O$ (g)	pH
1:1	0.666	7.8	250	0.174	0.174	7.0
1:2	0.666	8.0	250	0.087	0.087	7.2
1:3.2	0.666	8.2	250	0.0535	0.0535	10.8
1:6.5	0.666	8.2	250	0.0267	0.0267	10.9
1:13	0.666	8.3	250	0.0134	0.0134	10.7

Table S4. The parameters of the synthesis for the set of gels grafted with APTES (**APTES**:**SmS 1:10**).

APTES:SmS	$Na_2SiO_3·5H_2O$ (g)	DI H_2O (mL)	ETID (μL)	HCl 37% (μL)	APTES 98% (μL)	pH
1:10	0.666	9.33	250	340	74.8	6.90

Table S5. The parameters of the synthesis for the set of gels grafted with CPTS (**CPTS**:**SmS 1:10**).

CPTS:SmS	$Na_2SiO_3·5H_2O$ (g)	DI H_2O (mL)	ETID (μL)	HCl 37% (μL)	CPTS 97% (μL)	pH
1:10	0.666	9.37	250	340	57.5	6.90

Table S6. The parameters of the synthesis for the set of gels grafted with TESPSA (**TESPSA**:**SmS 1:20**).

TESPSA:SmS	$Na_2SiO_3·5H_2O$ (g)	DI H_2O (mL)	ETID (μL)	HCl 37% (μL)	TESPSA 97% (μL)	pH
1:20	0.666	9.37	250	340	44.0	6.90

Table S7. Data collected during the release for the quadruplets of Gel No 3 (“**Control**).

Time (hours)	Gel (No 3) 1st	Gel (No 3) 2nd	Gel (No 3) 3rd	Gel (No 3) 4th	Average $\bar{x}$	Standard Deviation (σ)
0	0	0	0	0	0	0
1	19.2493	22.1731	20.8437	20.8691	20.7838	1.036304593
2	31.0292	33.4791	32.8395	32.9844	32.58305	0.9279283175
3	39.1702	41.424	41.3373	42.3835	41.07875	1.175901923
4	46.7263	49.641	47.581	49.0714	48.254925	1.159601723
5	52.5698	54.7175	52.9942	51.8793	53.0402	1.046949887
6	56.2922	58.9242	57.1054	57.9533	57.568775	0.9784421965
9	61.5752	65.3814	64.3014	65.9682	64.30655	1.686466913
12	66.9909	69.9156	69.0959	69.2822	68.82115	1.099510178
24	71.7947	73.0737	72.8659	73.4524	72.796675	0.6155209435
30	71.4956	74.6469	74.3018	73.2177	73.4155	1.227498931
48	75.7635	75.4154	74.7668	74.681	75.156675	0.5085209994

Table S8. Initial Rate and % Final Release for Ca^{2+}-loaded hydrogels compared to the control gel.

Gel	Initial rate (μmole/min) 1st hour	Initial rate (μmole/min) 2nd hour	Initial rate (μmole/min) 3rd hour	Average Initial rate (μmole/min)	% Final Release
<u>Control (No 3)</u>	<u>4.16</u>	<u>4.11</u>	<u>2.74</u>	<u>3.67</u>	<u>74.7</u>
Ca:**ETID** 1:4	3.65	2.92	2.57	3.05	71.2
Ca:**ETID** 1:2	3.32	2.56	1.93	2.60	64.7

Table S9. Rates and % Final Release of Cu^{2+}-loaded hydrogels compared to the control gel.

Gel	Initial rate (μmole/min) 1st hour	Initial rate (μmole/min) 2nd hour	Initial rate (μmole/min) 3rd hour	Average Initial rate (μmole/min)	% Final Release
**Control (No 3)**	4.16	4.11	2.74	3.67	74.7
Cu:**ETID** 1:13	2.69	2.00	1.67	2.12	58.3
Cu:**ETID** 1:6.5	1.84	1.33	1.09	1.42	33.6
Cu:**ETID** 1:3.2	1.54	1.00	0.76	1.10	24.5
Cu:**ETID** 1:2	0.68	0.76	0.71	0.72	19.8
Cu:**ETID** 1:1	0	0	0	0	0

Table S10. Initial Rates and % Final Release of **ETID** from Ca^{2+}- and Cu^{2+}-loaded hydrogels.

Gel	Initial rate (μmole/min) 1st hour	Initial rate (μmole/min) 2nd hour	Initial rate (μmole/min) 3rd hour	Average Initial rate (μmole/min)	% Final Release
**No 3 (Control)**	4.16	4.11	2.74	3.67	**74.7**
**Ca^{2+}:ETID 1:2**	3.65	2.56	1.93	2.60	64.7
**Cu^{2+}:ETID 1:2**	0.68	0.76	0.71	0.72	19.6

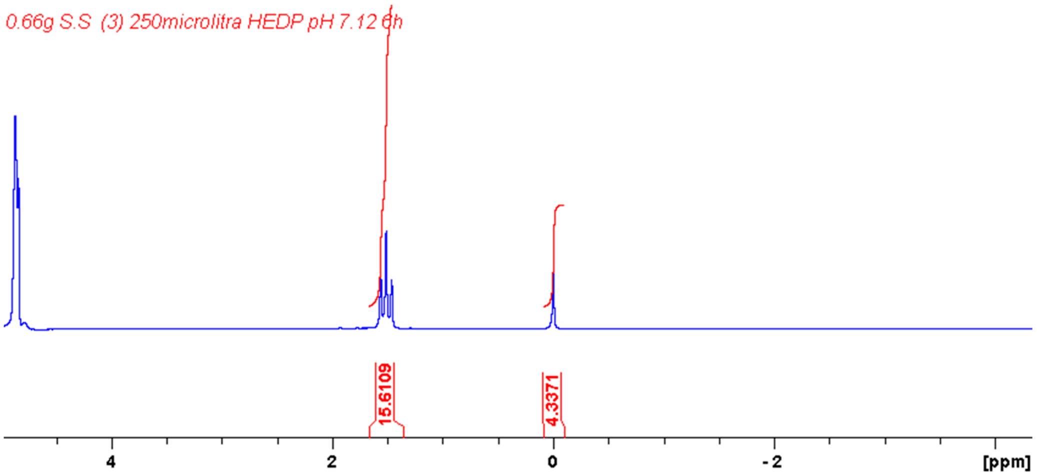

Figure S1. ^1H NMR of the aliquot (6th hour) withdrawn from the supernatant of Gel No 3 during the release experiment. Integration was performed to quantify the amount of **ETID** released to the supernatant phase.

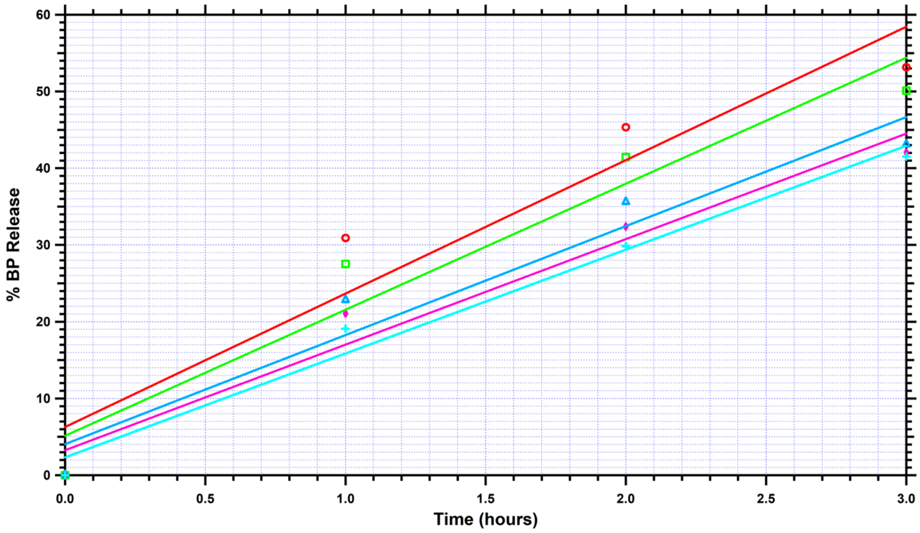

Figure S2. Normalization on the first three hours average % ETID Release of the five silica hydrogels. These are 3.3% (red), 4.0% (light green), 6.6% (blue), 13.3% (magenta) and 16.0% (turquoise) $^{w}/_{w}$ SmS in Water. SmS = sodium metasilicate.

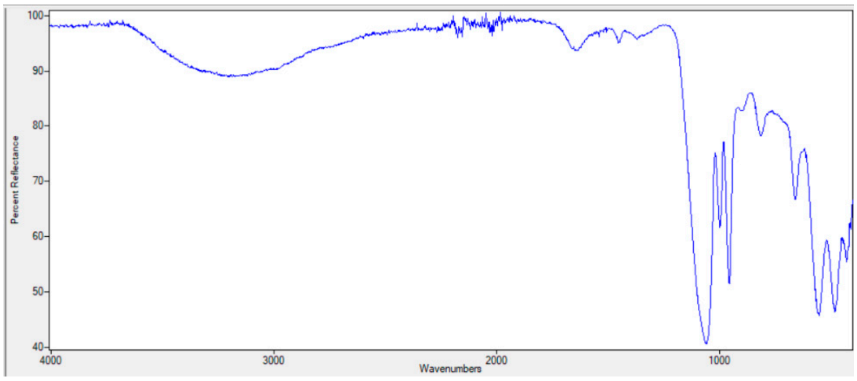

Figure S3. FTIR spectrum of the Calcium-ETID precipitate on top of the Calcium-drug-loaded hydrogel.

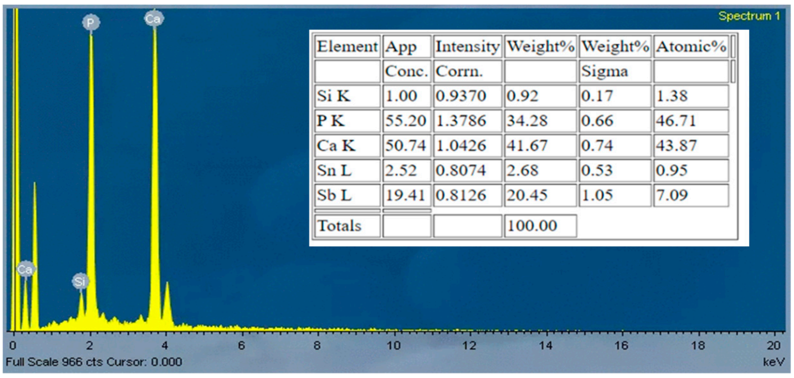

Element	App Conc.	Intensity Corrn.	Weight%	Weight% Sigma	Atomic%
Si K	1.00	0.9370	0.92	0.17	1.38
P K	55.20	1.3786	34.28	0.66	46.71
Ca K	50.74	1.0426	41.67	0.74	43.87
Sn L	2.52	0.8074	2.68	0.53	0.95
Sb L	19.41	0.8126	20.45	1.05	7.09
Totals			100.00		

Figure S4. EDX spectrum of the Calcium-ETID precipitate.

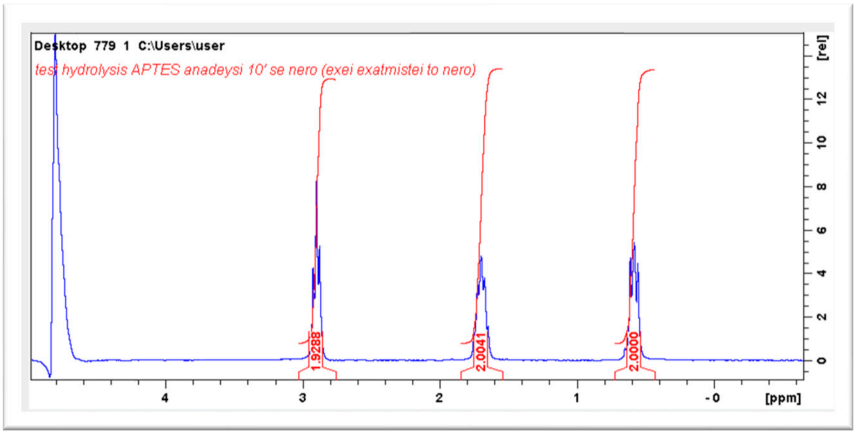

Figure S5. ^{1}H NMR data collected 10 minutes after the addition of APTES in water.

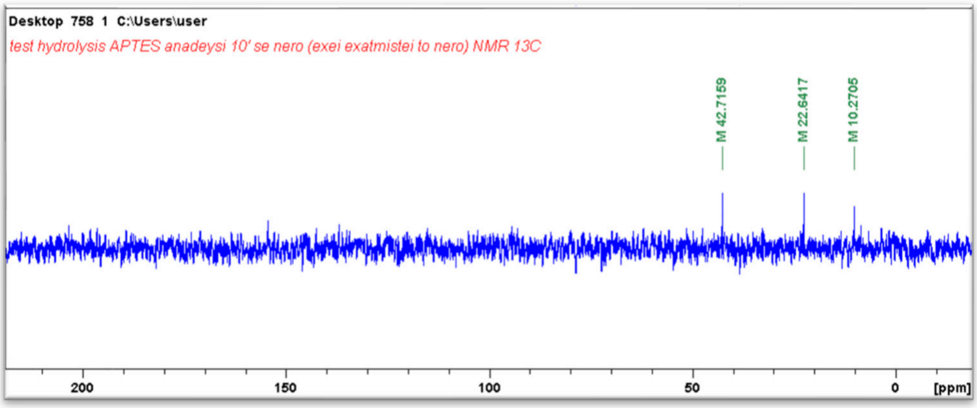

Figure S6. ^{13}C NMR data collected 10 minutes after the addition of APTES in water.

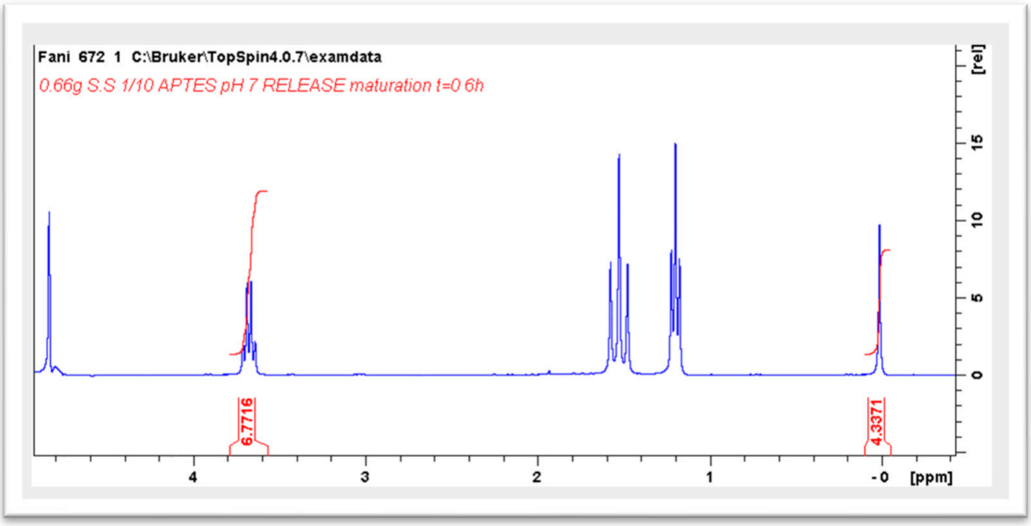

Figure S7. An example of ^{1}H NMR data collected (6th hour) from an APTES-ETID gel. The presence of ethanol peaks indicates the hydrolysis of the Si-O-CH_2CH_3 portion of APTES.

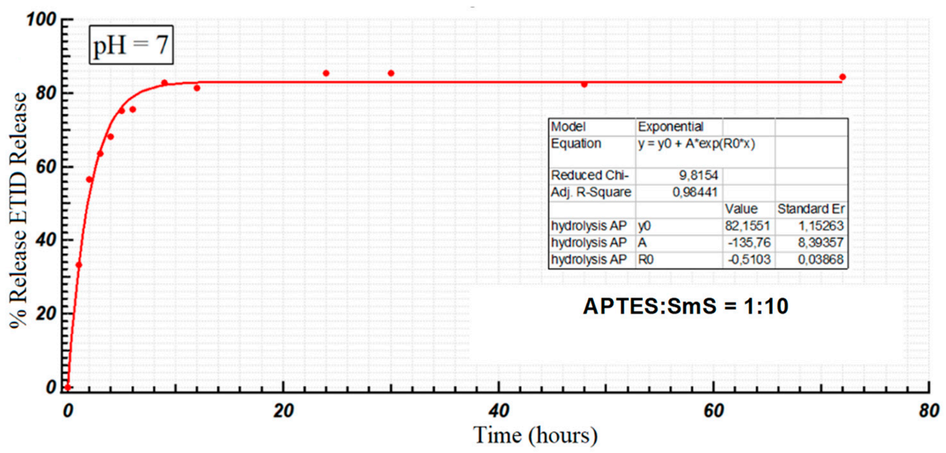

Figure S8. The "Release" Curve of Ethanol. The data indicate that 85% of APTES was hydrolyzed. SmS = sodium metasilicate.

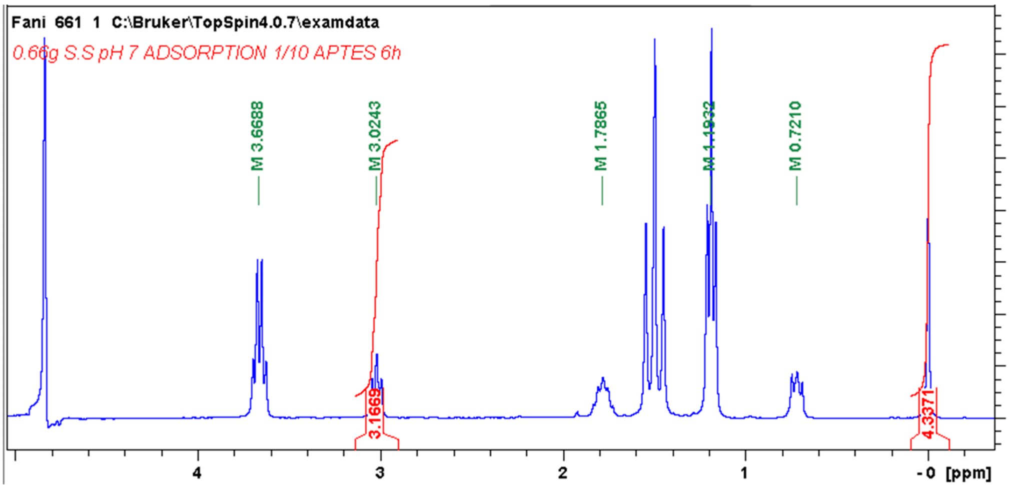

Figure S9. Monitoring of the APTES absorption from the supernatant and into the hydrogel.

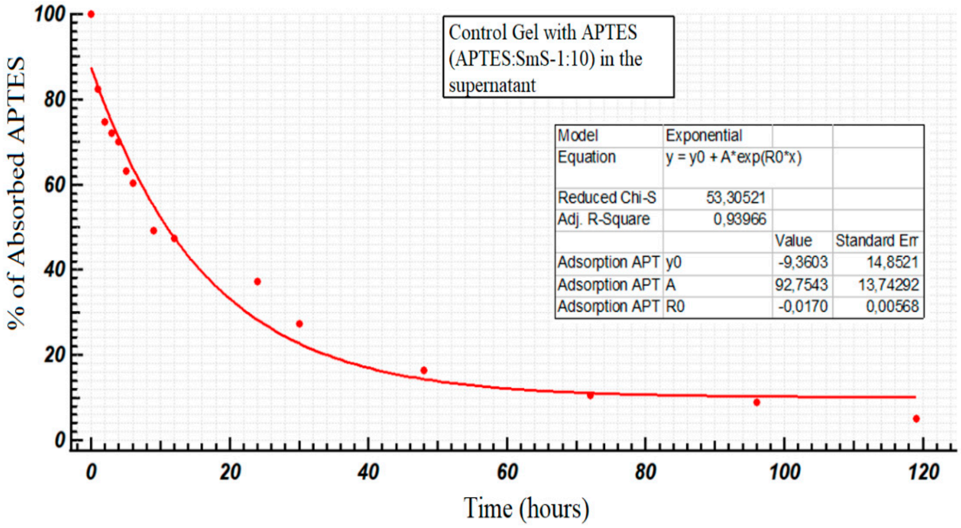

Figure S10. Absorption curve of APTES from the supernatant and into the hydrogel.

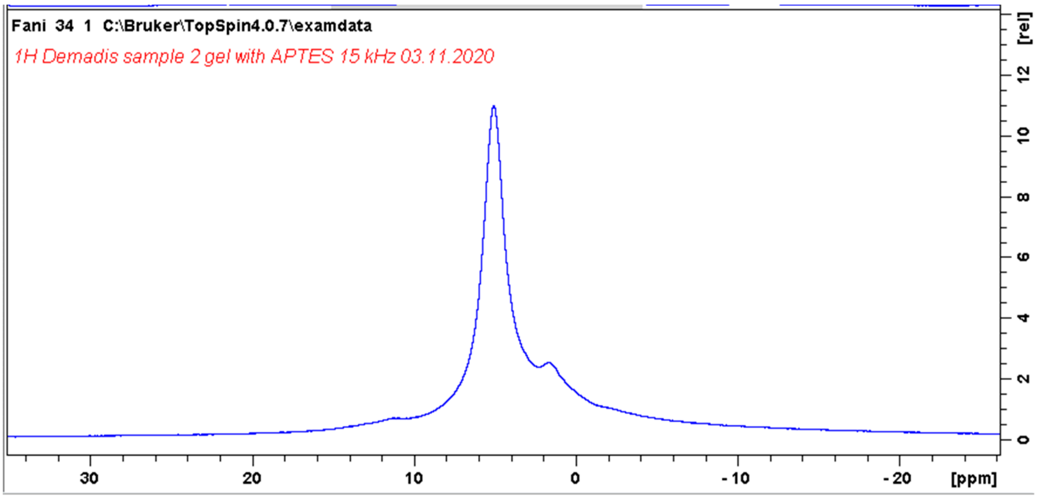

Figure S11. ^{1}H Solid State NMR data spectrum of an APTES-grafted hydrogel. The signal at 0.72 ppm indicates the presence of APTES in the hydrogel.

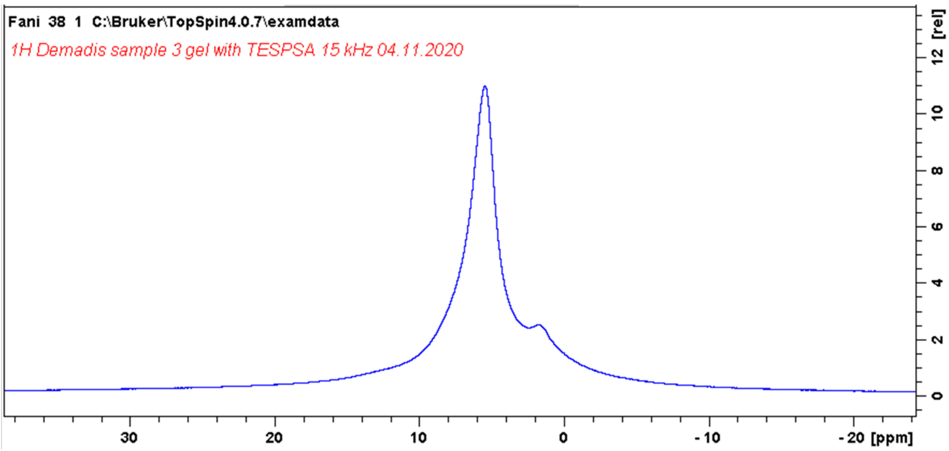

Figure S12. ^{1}H Solid State NMR data spectrum of TESPSA-grafted hydrogel. The signal at 0.72 ppm indicates the presence of TESPSA in the hydrogel.

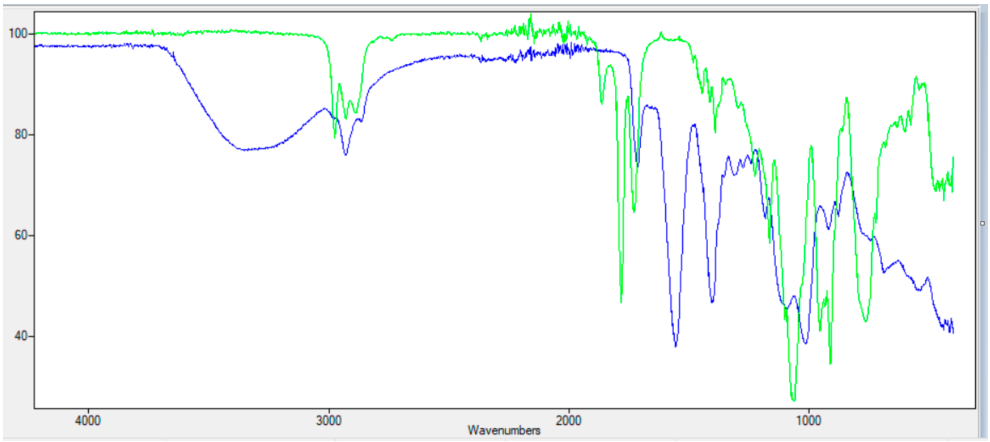

Figure S13. FT-IR of TESPA collected after the evaporation of the solvent. The spectrum of the "opened" succinic ring appears in blue and the spectrum of **TESPSA** is in green.

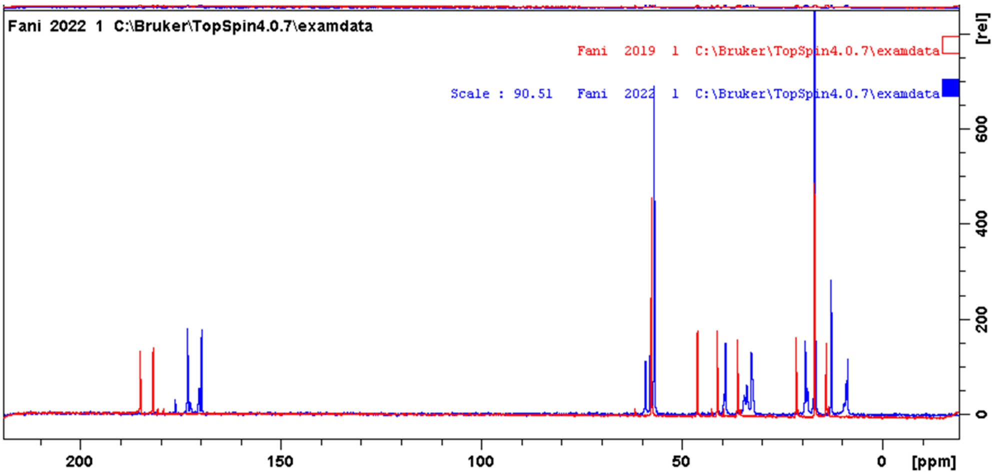

Figure S14. ^{13}C NMR spectra: (blue) pristine **TESPSA** reagent, (red) aqueous solution of **TESPSA** at pH~6,9 (red).
